# The Role of miR-640: A Potential Suppressor in Breast Cancer *via *Wnt7b/β-catenin Signaling Pathway

**DOI:** 10.3389/fonc.2021.645682

**Published:** 2021-04-12

**Authors:** Chun Tang, Xuehui Wang, Changle Ji, Wenfang Zheng, Yunhe Yu, Xiaochong Deng, Xiqian Zhou, Lin Fang

**Affiliations:** ^1^ Clinical Medical College of Shanghai Tenth People’s Hospital, Nanjing Medical University, Nanjing, China; ^2^ Department of Thyroid and Breast Surgery, Taizhou Fourth People’s Hospital, Taizhou, China; ^3^ Department of Thyroid and Breast Surgery, Shanghai Tenth People’s Hospital, School of Medicine, Tongji University, Shanghai, China

**Keywords:** miR-640, Wnt7b, β-catenin, Wnt/β-catenin pathway, breast cancer

## Abstract

In this study, we demonstrated that miR-640 is significantly downregulated in breast cancer (BC) tissues and cell lines. Overexpression of miR-640 inhibited the proliferation and migration of BC *in vitro* and *in vivo*, while depletion of miR-640 exhibited the opposite effect. Importantly, miR-640 could directly target Wnt7b, thereby regulating Wnt/β-catenin signaling pathway in BC. In conclusion, miR-640/Wnt7b suppresses BC cells tumorigenesis *via* Wnt/β-catenin signaling pathway, which might be novel targets for BC targeted therapy.

## Introduction

Breast cancer (BC) is well recognized as the most frequently diagnosed cancer in women worldwide with high incidence and mortality rates (1). Despite major breakthroughs in therapy strategies and therapeutic modalities of BC, the mortality rates of BC remain high due to metastasis and recurrence. Thus studies focusing on the mechanism of pathogenesis and metastasis of BC as well as new prognostic markers for precise are urgently needed.

Wnt signaling pathway, an important extracellular pathway, participates in a large set of cellular processes, including cell proliferation, differentiation, migration and apoptosis (2). The Wnt signaling pathway mainly comprises canonical Wnt pathway and noncanonical Wnt pathway according to whether β‐catenin is affected (3). The canonical Wnt pathway is highly evolutionary conserved and mainly involves β-catenin. Previous studies have shown that aberrant activation of Wnt/β-catenin signaling pathway has a critical role in oncogenesis and development of various cancers, including BC (4, 5). Several Wnt proteins, such as Wnt1 (6, 7), Wnt3a (8), Wnt7a (9), Wnt7b (10) and Wnt9a (11) have been reported to initiate canonical Wnt/β-catenin signaling by combining with Frizzled (FZD) or low‐density‐lipoprotein receptor‐related proteins 5/6 (LRP5/6), leading to the disassembly of the β-catenin destruction complex and nuclear translocation of β-catenin. Then β-catenin could combine with T-cell factor/lymphoid enhancer-binding factor (TCF/LEF), thereby promoting transcription of Wnt target genes (3, 12, 13).

Wnt7b is highly-expressed in many cancers and aberrant Wnt7b expression contributes to the pathogenesis of several cancers, such as pancreatic adenocarcinoma, bladder cancer and osteosarcoma (10, 14, 15) (**Figure S1**). More importantly, high expression of Wnt7b is associated with aggressive clinicopathologic features and poor clinical outcome of BC patients (16), indicating it an independent prognostic biomarker in BC.

MicroRNAs (miRNAs) are a group of non-coding small RNA molecules that inhibit the expression of target mRNAs post-transcriptionally (17). Multiple miRNAs or miRNA families/clusters have been recognized play vital roles in BC. For example, miR-424 plays an anti-oncogenic role in BC by inhibiting CDK1 expression (18). MiR-135b could promote BC cell growth and disrupt the cell cycle by regulating LATS2 (19). Recent studies indicate that miR-640 could aggravate intervertebral disc degeneration *via* NF-κB and WNT signaling pathway (20). In addition, miR-640 suppresses the progression of HCC *via* HIF-1α signaling Pathway (21). However, the role of miR-640 in BC is still unknown.

In this study, we found miR-640 was downregulated in BC tissues and cell lines. In addition, miR-640 plays an anti-oncogenic role *in vitro* and *in vivo* by inhibiting BC cells proliferation and migration. More importantly, we demonstrated that Wnt7b is a direct target of miR-640, so that miR-640 could act as a suppressor in BC *via* canonical Wnt/β-catenin signaling pathway.

## Materials and Methods

### Clinical Tissue Samples

50 tumor tissues and their adjacent normal tissues were collected from BC patients who underwent operation in the Department of Breast and Thyroid Surgery of Shanghai Tenth People’s Hospital of Tongji University (Shanghai, China). Patients receiving chemotherapy or radiotherapy before surgery were excluded. This study was approved by Institutional Ethics Committees of Shanghai Tenth People’s Hospital. We have obtained informed consent from all patients. All tissue specimens were snap-frozen in liquid nitrogen immediately for further use.

### Cell Culture and Transfection

We obtained the human BC cell lines MDA-MB-231, BT549, MCF-7, SKBR3 and normal breast epithelial cell line MCF-10A from Chinese Academy of Sciences (Shanghai, China). All BC cell lines were cultured in Dulbecco’s Modified Eagle’s Medium (DMEM) (Gibco, USA) with 10% Fetal Bovine Serum (FBS) (Gibco, USA), penicillin (100 units/ml) and streptomycin (100 μg/ml) (Enpromise, China), and mycoplasma elimination reagent (Yeasen, China). MCF-10A were cultured in Mammary Epithelial Basal Medium (MEBM) (Cambrex, USA). All these cells were cultured at 37°C with 5% CO_2_. MiR-640 mimics, miR-640 inhibitor and non-specific miR-negative control (miR-640-NC) oligo were purchased from RiboBio (Guangzhou, China). The specific siRNA of Wnt7b (si-Wnt7b) and negative control (si-NC) were purchased from Generay (Shanghai, China). Hieff Trans™ Liposomal Transfection Reagent (Yeasen, China) was used for transfection according to the protocols.

### RNA Extraction and RT-qPCR

We extracted total RNA from frozen tissues and cultured cells using Trizol reagent (Invitrogen, Carlsbad, CA, USA). The concentration and purity of RNA was assessed by Nanodrop 2000 spectrophotometer (Thermo Fisher Scientific, USA). We conducted quantitative real-time polymerase chain reaction (RT-qPCR) by using the Hieff^®^ qPCR SYBR^®^ Green Master Mix (Yeasen, China). Primer sequences were designed and synthesized by RiboBio (Guangzhou, China). Expression of miRNAs and mRNAs were assessed by threshold cycle (CT) values and analyzed using the 2^-ΔΔCt^ method. U6 and β-actin were used as internal control. Primers used in this study were shown in **Table S1**.

### MTT Assay

3-(4,5-dimethylthiazol-2-yl)-2,5-diphenyltetrazolium bromide (MTT) assay was used to evaluate cell proliferation. Cells were seeded in 96-well plates at 1*10^4^ cells/ml after 24h transfection. 1, 2, 3, 4, 5 day later, 20 μl MTT (at a concentration of 5 mg/ml; Sigma Aldrich) was added per well, and cells were cultured at 37°C with 5% CO_2_ for 4-6h. 150 μl DMSO was added after the discarding supernatant. The OD _490_ nm optical density was detected by a microplate reader (BioTek, USA).

### Colony Formation Assay

Transfected BC cells were seeded into 6-well plates (1000 cells per well). After 2 weeks, cell colonies were gently washed with cold 1 x PBS, fixed with 75% ethanol and stained with 0.1% crystalline purple. Then colonies were photographed and counted by Image J.

### Wound Healing Assay

Transfected BC cells were seeded in 12-well plates. When the cells reached more than 90% confluent, we produced a scratch by using a 200-μl-pipette tip over the surface of cells. Then cells were cultured with DMEM medium with 2%FBS. The scratch area was observed and measured under the microscope (Leica Microsystems, Mannheim, Germany) at 0h and 24h. Image J was used to measure the scratch area.

### Migration Assay

We used transwell chambers (Corning, Inc., Lowell, MA, USA) to measure the migration ability of MDA-MB-231 cells in 24-well plates. Transfected cells were placed in the upper chamber with 200 μl serum-free medium and medium with 10% FBS was added in the lower chamber. 14h later, noninvasive cells in the upper chamber were removed by cotton swabs and invasive cells on the opposite side were fixed with 95% ethanol for 10 min, stained with 0.1% crystal violet for 10 min. Pictures of invasive cells were taken with a microscope (Leica Microsystems, Mannheim, Germany) and migrated cells were counted in 3 randomly-selected fields.

### Luciferase Reporter Assay

Wild-type and mutant-type reporter plasmids of Wnt7b 3’-UTR were designed and synthesized by IBSBio (Shanghai, China). These two reporter plasmids were co-transected with miR-640 mimics or miR-640-NC into HEK293T cells. After 48h, luciferase activities were measured by the Dual-Luciferase^®^ Reporter Assay kit (Yeasen, China).

### FISH Assay

Specific probes for miR-640 were designed and synthesized by IBSBio (Shanghai, China). 4’,6-Diamidino-2-Phenylindole (DAPI) was used to stain cell nuclei. Ribo™ Fluorescent In Situ Hybridization Kit (Ribo, China) was used in FISH assay. Fluorescence microscope (Olympus BX53 Biological Microscope) was used to capture the images of cells.

### Western Blotting Assay

Proteins were isolated with RIPA lysis buffer (Beyotime, Jiangsu, China) after 48h of transfection. Protein lysates were separated by 10% sodium dodecyl sulfate-polyacrylamide gels and then transferred to nitrocellulose membrane (Beyotime, Jiangsu, China). Nitrocellulose membrane with proteins were immunoblotted overnight at 4°C with primary antibodies: anti-PCNA (Proteintech, USA), anti-Wnt7b (Proteintech, USA), β-catenin (Proteintech, USA), Gsk-3β (Proteintech, USA), cyclin D1 (Abcam, USA), C-myc (Wanlei, China). Subsequently, the membranes were incubated in secondary antibodies for 1h at room temperature. Dilutions of all antibodies used in this study were 1:1000. Odyssey Infrared scanning system (Li-Cor, Lincoln, NE, USA) was used to visualize protein bands.

### Xenograft Tumor Assay

Athymic nude mice (age, 4–6 weeks; weight, 18–22 g) were obtained from the laboratory animal center of Shanghai and maintained in specific pathogen-free (SPF) conditions. Nude mice were randomly divided into two groups of 4 each. Approximately 1 × 10^6^ MDA-MB-231 cells with stable expression of miR-640 or miR-640-NC were injected into the second mammary fat of the mice. The growth of tumors was monitored every 1 week according to the following formula: Volume (mm^3^) = 0.5 * width^2^ * length. After 7 weeks, the mice were sacrificed and the tumors were collected. The animal procedure was approved by the ethics committee of Tongji University.

### Immunohistochemistry (IHC)

IHC were performed on formalin-fixed, paraffin-embedded sections of fresh tumor tissue samples from the nude mice. The paraffin-embedded tissue was incubated with anti-Wnt7b antibody (Proteintech, USA) at 1:250 dilution to measure Wnt7b expression overnight at 4°C. Images were captured under a microscope (Leica Microsystems, Mannheim, Germany) at the appropriate magnification.

### Statistical Analysis

The significance of differences between groups from three independent experiments was assessed by GraphPad Prism (GraphPad, CA, USA). All data were presented as the means ± standard deviation (SD). The Student’s t-test was used for comparison between groups and P-value < 0.05 was considered statistically significant.

## Results

### MiR-640 Was Down-Regulated in BC Cell Lines and Tissues

**Figure 1:**
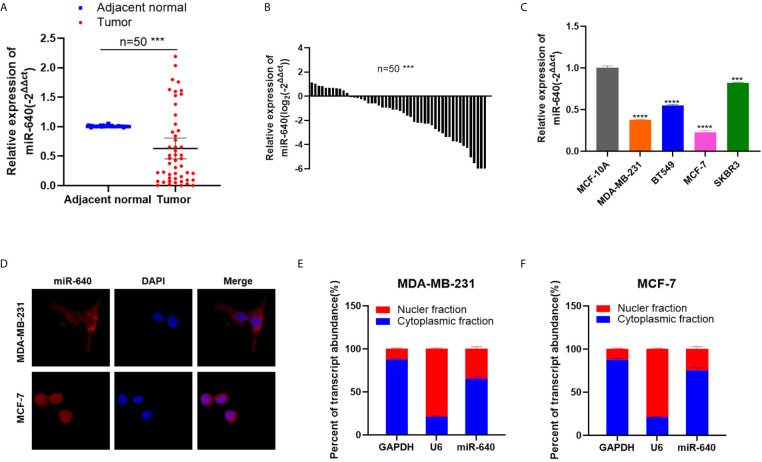
MiR-640 was down-regulated in BC cell lines and tissues. **(A, B)** MiR-640 had low expression in BC tissues compared with adjacent normal tissues. **(C)** MiR-640 had low expression in BC cell lines. **(D)** Detection of colocalization of miR-640 in cytoplasm by FISH assay (magnification, × 400). Red, miR-640; Blue, DAPI. **(E, F)** Expression levels of cytoplasmic control transcripts (GAPDH), the nuclear control transcript (U6), and miR-640 were determined by RT-qPCR in the cytoplasmic and nuclear fractions of BC cells. ***p < 0.001; ****p < 0.0001.

We searched several cancer research databases for characterizing the expression of miR-640, but there is no related report over the expression of miR-640 in BC. Thus, we first assessed the expression of miR-640 by RT-qPCR in 50 pairs of BC tissues and adjacent normal tissues. Our results showed that the expression of miR-640 was significantly downregulated in BC tissues (35/50, 70%) (**Figures 1A, B**). Additionally, we found the expression of miR-640 was lower in BC cell lines than in MCF-10A, especially in basal-like cohort and luminal cohort (**Figure 1C**). Then, we performed FISH assay to detected the localization of miR-640 and revealed that miR-640 was mostly stained in cytoplasm of MDA-MB-231 and MCF-7 (**Figure 1D**). Results of subcellular fractionation further verified the above results (**Figures 1E, F**). To better verify the role of miR-640 in BC, we analyzed the relationship between the expression of miR-640 and the clinical pathological variables in 50 BC patients. As shown in **Table 1**, high expression of miR-640 was negatively associated with TNM stage, tumor size and distant metastasis, but had no correlation with age and lymph node metastasis.

**Table 1:** The relationship between the expression of miR-640 and various clinicopathological variables.

Patients Characteristics	Total	miR-640 expression		P value*
		High (*N=15)*	Low (*N=35)*	
Age				0.2077
<60	20	8	12	
≥60	30	7	23	
TNM stage				0.0434*
I/II	33	13	20	
III/IV	17	2	15	
Tumor size(cm)				0.0009***
≤2	29	14	15	
>2	21	1	20	
Lymph node metastasis				0.0588
negative	30	12	18	
positive	20	3	17	
Distant metastasis				0.0434*
No	42	15	27	
Yes	8	0	8	

P value from Chi-square test (*P < 0.05，*** P < 0.001.)

### MiR-640 Suppressed Cell Proliferation of BC Cells

**Figure 2:**
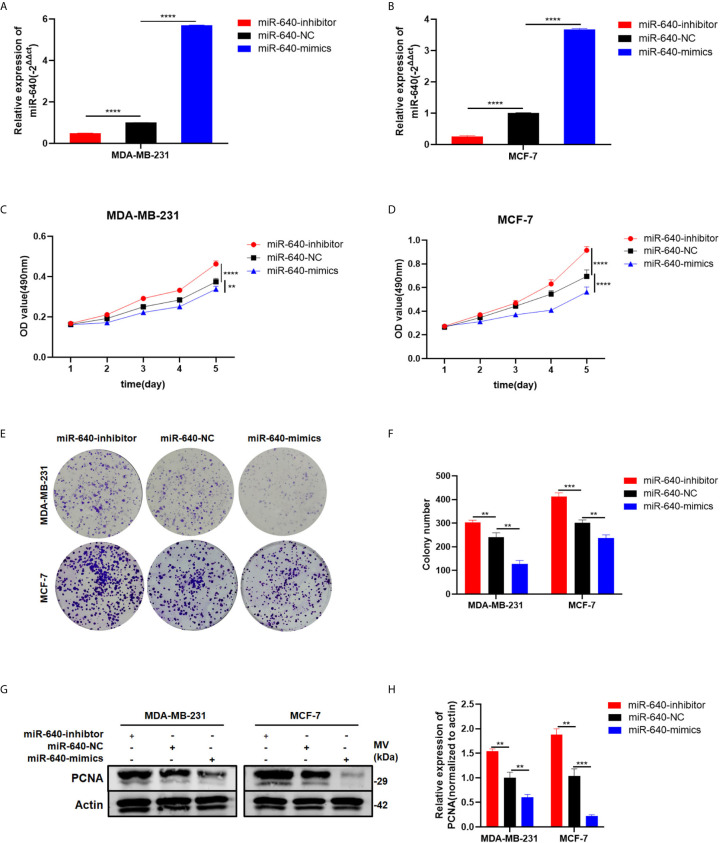
MiR-640 suppressed cell proliferation of BC cells. **(A, B)** Expression of miR-640 was confirmed by RT-qPCR in MDA-MB-231 and MCF-7 cells. **(C, D)** Effect of miR-640 on proliferation in MDA-MB-231 and MCF-7 cells by MTT assay. **(E, F)** Effect of miR-640 on proliferation in MDA-MB-231 and MCF-7 cells by colony formation assay. **(G, H)** Effect of miR-640 on proliferation in MDA-MB-231 and MCF-7 cells by western blotting. **p < 0.01; ***p < 0.001; ****p < 0.0001.

The transfection efficiency of miR-640-mimics and miR-640-inhibitor were verified by RT-qPCR (**Figures 2A, B**). Then, the MTT assay and colony formation assay revealed that upregulation of miR-640 caused a significant decrease in BC cells viability relative to the control group (**Figures 2C, F**). Meanwhile, the expression of proliferation marker PCNA was inhibited by miR-640-mimics demonstrated by western blotting (**Figures 2G, H**). All results above indicated that miR-640 act as an anti-tumor miRNA in BC.

### MiR-640 Suppressed Cell Migration of BC Cells

**Figure 3:**
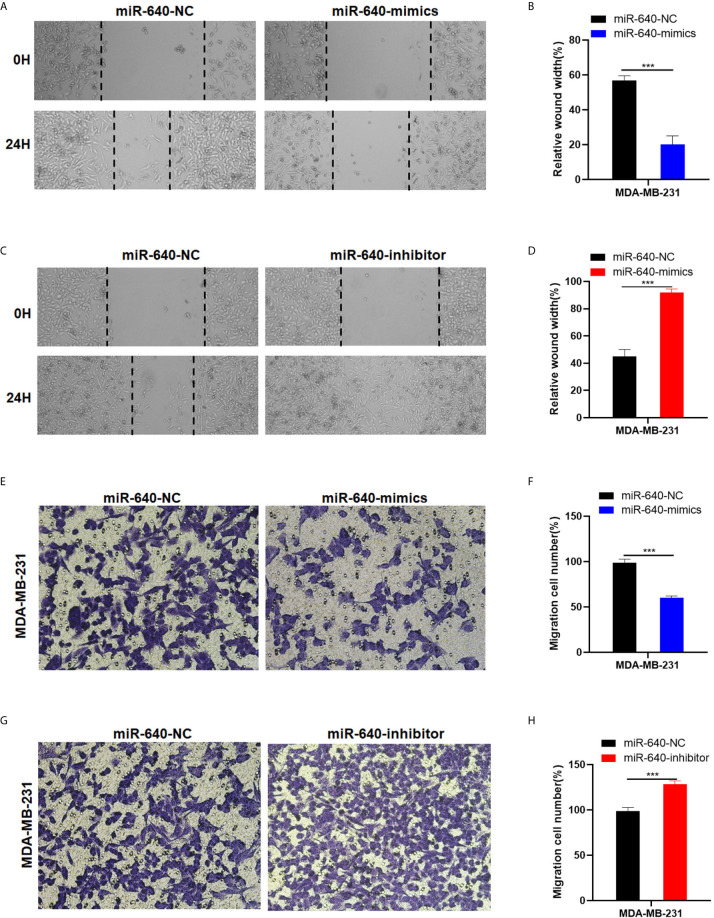
MiR-640 suppressed cell migration of BC cells. **(A–D)** Wound healing assays were performed in MDA-MB-231 cell line treated with miR-640-mimics or miR-640-inhibitor (miR-NC as negative control). **(D–H)** Cell migration assays were performed in MDA-MB-231 cell line treated with miR-640-mimics or miR-640-inhibitor (miR-NC as negative control). ***p < 0.001.

The wound healing assay and transwell migration assay were performed to determine whether miR-640 affect BC cells migration. The results showed that the scratched area healing rate of the miR-640-mimics group was smaller compared to miR-640-NC group after 24 hours (**Figures 3A, B**). Consistently, we found that elevated miR-640 decreased the number of cells that passed through the membranes of transwell chamber in MDA-MB-231cell line *via* transwell migration assay (**Figures 3E, F**). The miR-640-inhibitor group showed the opposite results (**Figures 3C, D, G, H**). These results indicated that miR-640 inhibit the migration of MDA-MB-231 cells.

### Wnt7b Is a Direct Target of miR-640

**Figure 4:**
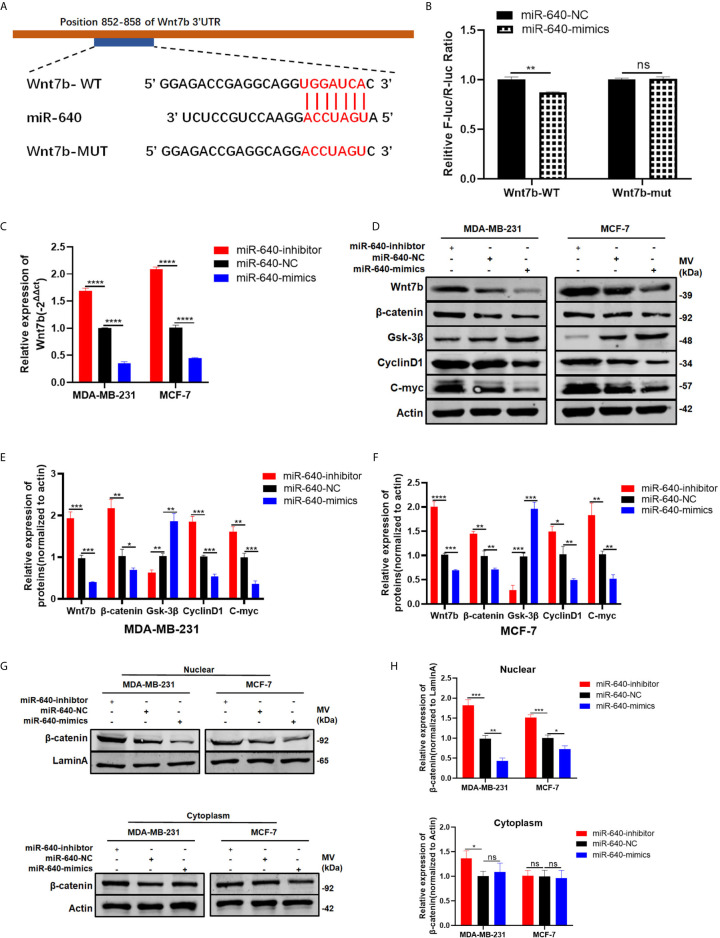
Wnt7b is a direct target of miR-640. **(A)** Putative complementary site within miR-640 and Wnt7b predicted by bioinformatics analysis (TargetScan). **(B)** Luciferase reporter assay demonstrated that Wnt7b is a direct target of miR-640. **(C)** Wnt7b mRNA level was determined by RT-PCR in MDA-MB-231 and MCF-7 cells with different treatment. **(D–F)** Representative Western blotting and quantification of Wnt7b, β-catenin, Gsk-3β, Cyclin D1 and C-myc in MDA-MB-231 and MCF-7 cells with different treatment, β-actin was used as a control. **(G, H)** Representative Western blotting and quantification of β-catenin in nuclear fraction and cytoplasm fraction of BC cells with different treatment, LaminA was used as a control. *p < 0.05; **p < 0.01; ***p < 0.001; ****p < 0.0001. "ns" means not statistically significant and represents P < 0.05.

Wnt7b was predicted have a direct binding site with miR-640 according to Targetscan (**Figure 4A**). Thus, luciferase reporter assay was performed to verify that Wnt7b is the direct target gene of miR-640. We constructed Wnt7b 3′-UTRs plasmids containing wild-type and mutant-type miR-640 binding sites. Through co-transfection of luciferase reporter plasmids and miR-640-mimics or miR-640-NC, we verified that wild-type Wnt7b 3′-UTR luciferase activity was remarkably decreased upon miR-640-overexpression (**Figure 4B**). After confirming Wnt7b is a direct target of miR-640, we further validated the effects of miR-640 on Wnt7b. Wnt7b expression in miR-640-overexpression or miR-640-depletion MDA-MB-231 and MCF-7 cells were determined by RT-qPCR. The results showed that the mRNA level of Wnt7b was remarkably downregulated *via* miR-640-overexpression while upregulated *via* miR-640-depletion (**Figure 4C**). Consistently, the results of western blotting indicated that miR-640 overexpression reduced Wnt7b, β-catenin, C-myc, and cyclin D1 protein levels whereas increased Gsk-3β protein level (**Figures 4D–F**). The miR-640 depletion showed the opposite results. More importantly, miR-640 upregulation inhibited the expression of β-catenin in the nuclear fraction of BC cells (**Figures 4G, H**). The above results prompted us to explore whether miR-640/Wnt7b suppresses BC cell tumorigenesis *via* Wnt/β-catenin signaling pathway.

### Depletion of Wnt7b Rescued the Effect of miR-640-Inhibitor on BC Cells

**Figure 5:**
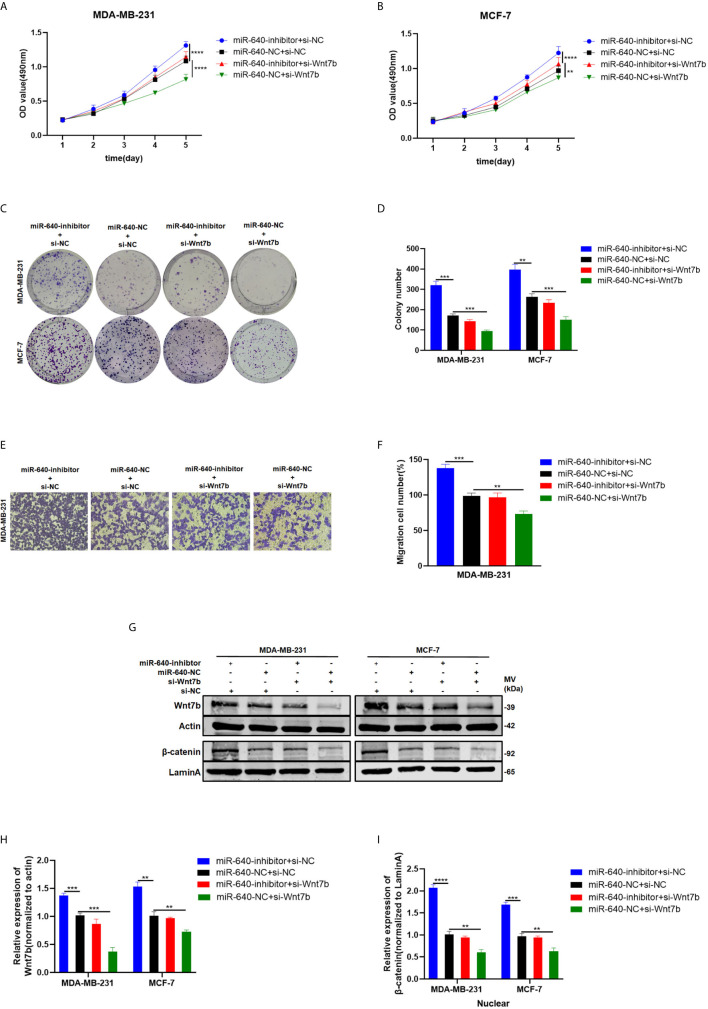
Depletion of Wnt7b rescued the effect of miR-640-inhibitor on BC cells. **(A–D)** Knockdown of Wnt7b partially reversed miR-640-inhibitor-induced promotion of proliferation in MDA-MB-231 and MCF-7 cells determined by MTT assay and colony assay. **(E, F)** Knockdown of Wnt7b partially reversed miR-640-inhibitor-induced promotion of migration in MDA-MB-231 and MCF-7 cells determined by transwell assay. **(G–I)** Western blotting analysis for Wnt7b/β-catenin protein level in MDA-MB-231 and MCF-7 cells. **p < 0.01, ***p < 0.001, ****p < 0.0001.

We designed rescue assays to further determine whether miR-640 affects the proliferation and migration of BC cells *via* Wnt7b. MDA-MB-231 and MCF-7 cells were co-transfected with miR-640-inhibitor and si-Wnt7b. Based on the results in **Figure 5**, the promotive effect of miR-640-inhibitor on BC cells was partially reversed by Wnt7b silence (**Figures 5A–F**). Consistently, the effect of miR-640-inhibitor on Wnt7b protein level was also partially reversed by si-Wnt7b (**Figures 5G, H**). More importantly, expression of β-catenin in the nuclear fraction showed the same changes with Wnt7b (**Figures 5G, I**).

### Overexpression of miR-640 Inhibited BC Growth *In Vivo*

**Figure 6:**
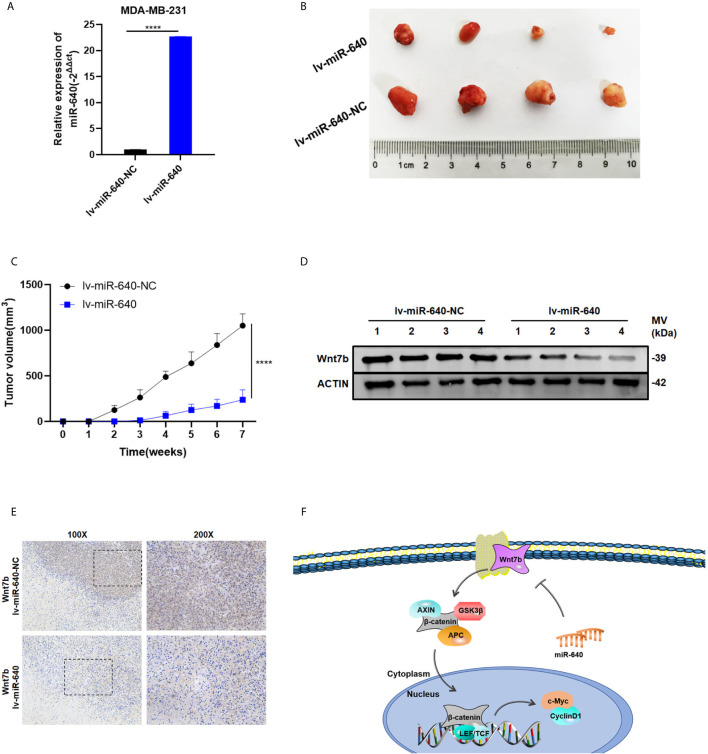
Overexpression of miR-640 inhibited BC growth *in vivo*. **(A)** Overexpression of miR-640 in MDA-MB-231 cells was verified by RT-qPCR. **(B)** Representative images of xenograft tumors in nude mice. **(C)** The growth curves of xenografts. **(D)** Extract protein from tumors and measuring the expression of Wnt7b by Western blotting. **(E)** Immunohistochemistry (IHC) staining of Wnt7b in xenografts. **(F)** The mechanism diagram was generated to illustrate the mechanism of miR-640/Wnt7b/Wnt-β-catenin axis in BC. ****p < 0.0001.

We established the MDA-MB-231 cell xenograft model to verify the effect of miR-640 in BC *in vivo*. As shown in **Figure 6A**, MDA-MB-231 cells were stably infected by lentiviral(lv-miR-640 or lv- miR-640-NC). 7 weeks later, tumors from the nude mice were collected and measured. Obviously, tumors in the miR-640-overexpresssion group were significantly smaller than those in miR-640-NC group, indicating that tumor growth was significantly inhibited by the miR-640 (**Figures 6B, C**). Then proteins were extracted from mice tumors and western blotting results indicated that the protein level of Wnt7b decreased in the miR-640-overexpression group (**Figure 6D**). Moreover, IHC analysis further elucidated that tumors of miR-640-overexpression had lower Wnt7b expression (**Figure 6E**). Taking all results *in vivo* and *in vitro* together, we confirmed that miR-640/Wnt7b suppresses BC cells tumorigenesis *via* Wnt/β-catenin signaling pathway. The mechanism was generated in **Figure 6F**.

## Discussion

As a group of small non-coding RNAs, miRNAs play key roles in the gene regulatory network of tumors by binding to the 3’ untranslated region (3’-UTR) of target mRNAs to inhibit gene expression. In recent years, multiple studies have focused on the function and mechanism of miRNAs in BC, so that many miRNAs have been found related to the initiation and progression of BC. Additionally, miRNA−based therapies have already been used as vital strategies in BC (22). In recent years, several studies revealed that miR-640 participate in numerous different biological processes. MiR-640 could promote Kupffer cells inflammation *via* restraining LRP1 and Wnt signaling pathway, indicating it a potential target for the therapy of acute liver injury in the future (23). MiR-640 participates in the process of sevoflurane-induced abnormal cognition *via* regulating ZFP91 (24). In addition, miR-640 plays a pivotal role in mediating the proangiogenic effect of H2S (25). However, there is no related reports over the role of miR-640 in the tumorigenesis and progression of BC. This study is the first to show that the expression of miR-640 in BC and the first to report the mechanism and clinical significance of miR-640 in BC.

In the present study, we first investigated the expression of miR-640 in 50-paired clinical BC tissues and adjacent normal tissues, and revealed that miR-640 expression is dramatically downregulated in BC. Of note, high expression of miR-640 was negatively associated with TNM stage, tumor size and distant metastasis of BC. Consistent with its expression pattern, overexpression of miR-640 could remarkably inhibit the proliferation and migration of BC *in vitro* and *in vivo*. Based on the expression level and function of miR-640 in BC, we further hypothesized that miR-640 could directly inhibit Wnt7b according to according to prediction from Targetscan.

Wnt7b, an important member of the Wnt proteins family, have been reported highly-expressed in many malignant tumors, including BC (26–28). Wnt7b could activate mTORC1 *via* PI3K/AKT signaling pathway to promote bone formation (29). Moreover, Wnt7b promotes cancer cell androgen-independent growth by activating protein kinase C isozymes in advanced prostate cancer (26). Of note, Wnt7b have been reported facilitate several cancers tumorigenesis *via* regulating canonical Wnt/β-catenin signaling (28, 30, 31). To verify whether miR-640 could directly target Wnt7b, luciferase reporter assay was performed. Then, according to the results of RT-qPCR and western blotting, both mRNA and protein level of Wnt7b were negatively regulated by miR-640. Consistently, protein levels of total β-catenin, cyclinD1 and C-myc were negatively regulated by miR-640 while Gsk-3β showed opposite results. More importantly, miR-640 upregulation inhibited the expression of β-catenin in the nuclear fraction, indicating the inhibitory effect of miR-640 on canonical Wnt/β-catenin signaling pathway. Finally, the rescue experiment further verified miR-640 suppresses BC *via* Wnt7b/β-catenin signaling pathway.

In conclusion, our findings demonstrated that miR-640 is downregulated in BC tissues and cell lines, and is able to suppress BC tumorigenesis *in vitro* and *in vivo* through directly targeting Wnt7b and Wnt/β-catenin signaling pathway. Therefore, the miR-640/Wnt7b/β-catenin axis might be novel targets for BC targeted therapy.

## Data Availability Statement

The original contributions presented in the study are included in the article/**Supplementary Material**. Further inquiries can be directed to the corresponding author.

## Ethics Statement

The studies involving human participants were reviewed and approved by Ethics Committee of Shanghai Tenth People’s Hospital. The patients/participants provided their written informed consent to participate in this study. The animal study was reviewed and approved by Ethics Committee of Shanghai Tenth People’s Hospital.

## Author Contributions

CT, XW, and LF designed the research. CT and XW performed the research and analyzed results. XW wrote the paper. CJ, WZ, YY, XD, and XZ edited the manuscript and provided critical comments. All authors contributed to the article and approved the submitted version.

## Funding

This work was supported by National Natural Science Foundation of China (No. 82073204).

## Conflict of Interest

The authors declare that the research was conducted in the absence of any commercial or financial relationships that could be construed as a potential conflict of interest.

## Supplementary Material

Supplementary Figure 1 Expression of Wnt7b in BC (TCGA database).

## Abbreviations

BC, breast cancer; ncRNA, non-coding RNA; miRNA, microRNA; Wnt7b, wingless-type MMTV integration site family, member 7B; β-catenin, catenin beta 1; RT-Qpcr, Quantitative real-time polymerase chain reaction; FISH, Fluorescent *In Situ* Hybridization.

## References

[1] Bray F Ferlay J Soerjomataram I Siegel RL Torre LA Jemal A. Global cancer statistics 2018: GLOBOCAN estimates of incidence and mortality worldwide for 36 cancers in 185 countries. CA Cancer J Clin (2018) 68:394–424. 10.3322/caac.21492 30207593

[2] Baarsma HA Königshoff M Gosens R. The WNT signaling pathway from ligand secretion to gene transcription: molecular mechanisms and pharmacological targets. Pharmacol Ther (2013) 138:66–83. 10.1016/j.pharmthera.2013.01.002 23328704

[3] Yin P Wang W Zhang Z Bai Y Gao J Zhao C. Wnt signaling in human and mouse breast cancer: Focusing on Wnt ligands, receptors and antagonists. Cancer Sci (2018) 109:3368–75. 10.1111/cas.13771 PMC6215866 30137666

[4] King TD Suto MJ Li Y. The Wnt/β-catenin signaling pathway: a potential therapeutic target in the treatment of triple negative breast cancer. J Cell Biochem (2012) 113:13–8. 10.1002/jcb.23350 PMC10924801 21898546

[5] Yeo EJ Cassetta L Qian BZ Lewkowich I Li JF Stefater JA,3. Myeloid WNT7b mediates the angiogenic switch and metastasis in breast cancer. Cancer Res (2014) 74:2962–73. 10.1158/0008-5472.Can-13-2421 PMC4137408 24638982

[6] Si W Li Y Shao H Hu R Wang W Zhang K. MiR-34a Inhibits Breast Cancer Proliferation and Progression by Targeting Wnt1 in Wnt/β-Catenin Signaling Pathway. Am J Med Sci (2016) 352:191–9. 10.1016/j.amjms.2016.05.002 27524218

[7] Katoh M. Expression and regulation of WNT1 in human cancer: up-regulation of WNT1 by beta-estradiol in MCF-7 cells. Int J Oncol (2003) 22:209–12. 10.3892/ijo.22.1.209 12469206

[8] He Q Yan H Wo D Liu J Liu P Zhang J. Wnt3a suppresses Wnt/β-catenin signaling and cancer cell proliferation following serum deprivation. Exp Cell Res (2016) 341:32–41. 10.1016/j.yexcr.2015.11.025 26643293

[9] King ML Lindberg ME Stodden GR Okuda H Ebers SD Johnson A. WNT7A/β-catenin signaling induces FGF1 and influences sensitivity to niclosamide in ovarian cancer. Oncogene (2015) 34:3452–62. 10.1038/onc.2014.277 PMC4345161 25174399

[10] Arensman MD Kovochich AN Kulikauskas RM Lay AR Yang PT Li X. WNT7B mediates autocrine Wnt/β-catenin signaling and anchorage-independent growth in pancreatic adenocarcinoma. Oncogene (2014) 33:899–908. 10.1038/onc.2013.23 23416978 PMC3923845

[11] Später D Hill TP O’Sullivan R J Gruber M Conner DA Hartmann C. Wnt9a signaling is required for joint integrity and regulation of Ihh during chondrogenesis. Development (2006) 133:3039–49. 10.1242/dev.02471 16818445

[12] Alok A Lei Z Jagannathan NS Kaur S Harmston N Rozen SG. Wnt proteins synergize to activate β-catenin signaling. J Cell Sci (2017) 130:1532–44. 10.1242/jcs.198093 28289266

[13] Mills KM Szczerkowski JLA Habib SJ. Wnt ligand presentation and reception: from the stem cell niche to tissue engineering. Open Biol (2017) 7:170140. 10.1098/rsob.170140 28814649 PMC5577451

[14] Bui TD O’Brien T Crew J Cranston D Harris AL. High expression of Wnt7b in human superficial bladder cancer vs invasive bladder cancer. Br J Cancer (1998) 77:319–24. 10.1038/bjc.1998.49 PMC2151239 9461004

[15] Liu Q Wang Z Zhou X Tang M Tan W Sun T. miR-342-5p inhibits osteosarcoma cell growth, migration, invasion, and sensitivity to Doxorubicin through targeting Wnt7b. Cell Cycle (2019) 18:3325–36. 10.1080/15384101.2019.1676087 PMC6927707 31601147

[16] Chen J Liu TY Peng HT Wu YQ Zhang LL Lin XH. Up-regulation of Wnt7b rather than Wnt1, Wnt7a, and Wnt9a indicates poor prognosis in breast cancer. Int J Clin Exp Pathol (2018) 11:4552–61. PMC6962957 31949853

[17] Bertoli G Cava C Castiglioni I. MicroRNAs: New Biomarkers for Diagnosis, Prognosis, Therapy Prediction and Therapeutic Tools for Breast Cancer. Theranostics (2015) 5:1122–43. 10.7150/thno.11543 PMC4508501 26199650

[18] Xie D Song H Wu T Li D Hua K Xu H. MicroRNA−424 serves an anti−oncogenic role by targeting cyclin−dependent kinase 1 in breast cancer cells. Oncol Rep (2018) 40:3416–26. 10.3892/or.2018.6741 PMC6196586 30272324

[19] Hua K Jin J Zhao J Song J Song H Li D. miR-135b, upregulated in breast cancer, promotes cell growth and disrupts the cell cycle by regulating LATS2. Int J Oncol (2016) 48:1997–2006. 10.3892/ijo.2016.3405 26934863

[20] Dong W Liu J Lv Y Wang F Liu T Sun S. miR-640 aggravates intervertebral disc degeneration via NF-κB and WNT signalling pathway. Cell Prolif (2019) 52:e12664. 10.1111/cpr.12664 31343104 PMC6797513

[21] Zhai Z Fu Q Liu C Zhang X Jia P Xia P. Emerging Roles Of hsa-circ-0046600 Targeting The miR-640/HIF-1α Signalling Pathway In The Progression Of HCC. Onco Targets Ther (2019) 12:9291–302. 10.2147/ott.S229514 PMC6842743 31807009

[22] Gambari R Brognara E Spandidos DA Fabbri E. Targeting oncomiRNAs and mimicking tumor suppressor miRNAs: New trends in the development of miRNA therapeutic strategies in oncology (Review). Int J Oncol (2016) 49:5–32. 10.3892/ijo.2016.3503 27175518 PMC4902075

[23] Wang GX Pan JY Wang YJ Huang TC Li XF. MiR-640 inhibition alleviates acute liver injury via regulating WNT signaling pathway and LRP1. Eur Rev Med Pharmacol Sci (2020) 24:8988–96. 10.26355/eurrev_202009_22841 32964988

[24] Xu W Zhao Y Ai Y. Overexpression of lncRNA Gm43050 alleviates apoptosis and inflammation response induced by sevoflurane treatment by regulating miR-640/ZFP91. Am J Transl Res (2020) 12:4337–46. PMC7476152 32913509

[25] Zhou Y Li XH Zhang CC Wang MJ Xue WL Wu DD. Hydrogen sulfide promotes angiogenesis by downregulating miR-640 via the VEGFR2/mTOR pathway. Am J Physiol Cell Physiol (2016) 310:C305–317. 10.1152/ajpcell.00230.2015 26879375

[26] Zheng D Decker KF Zhou T Chen J Qi Z Jacobs K. Role of WNT7B-induced noncanonical pathway in advanced prostate cancer. Mol Cancer Res (2013) 11:482–93. 10.1158/1541-7786.Mcr-12-0520 PMC4141540 23386686

[27] Kirikoshi H Sekihara H Katoh M. Molecular cloning and characterization of human WNT7B. Int J Oncol (2001) 19:779–83. 10.3892/ijo.19.4.779 11562755

[28] Qiu JJ Sun SG Tang XY Lin YY Hua KQ. Extracellular vesicular Wnt7b mediates HPV E6-induced cervical cancer angiogenesis by activating the β-catenin signaling pathway. J Exp Clin Cancer Res (2020) 39:260. 10.1186/s13046-020-01745-1 33234148 PMC7687741

[29] Chen J Tu X Esen E Joeng KS Lin C Arbeit JM. WNT7B promotes bone formation in part through mTORC1. PloS Genet (2014) 10:e1004145. 10.1371/journal.pgen.1004145 24497849 PMC3907335

[30] Zhang C Yang X Fu C Liu X. Combination with TMZ and miR-505 inhibits the development of glioblastoma by regulating the WNT7B/Wnt/β-catenin signaling pathway. Gene (2018) 672:172–9. 10.1016/j.gene.2018.06.030 29906532

[31] de Mattos RM Pereira PR Barros EG da Silva JH Palmero CY da Costa NM. Aberrant levels of Wnt/β-catenin pathway components in a rat model of endometriosis. Histol Histopathol (2016) 31:933–42. 10.14670/hh-11-730 26853489

