# Supplementary figures and images for: The Role of miR-640: A Potential Suppressor in Breast Cancer via Wnt7b/β-catenin Signaling Pathway
Source: Front Oncol. 2021 Apr 12;11:645682. doi: 10.3389/fonc.2021.645682 (PMC8072343; doi:10.3389/fonc.2021.645682)

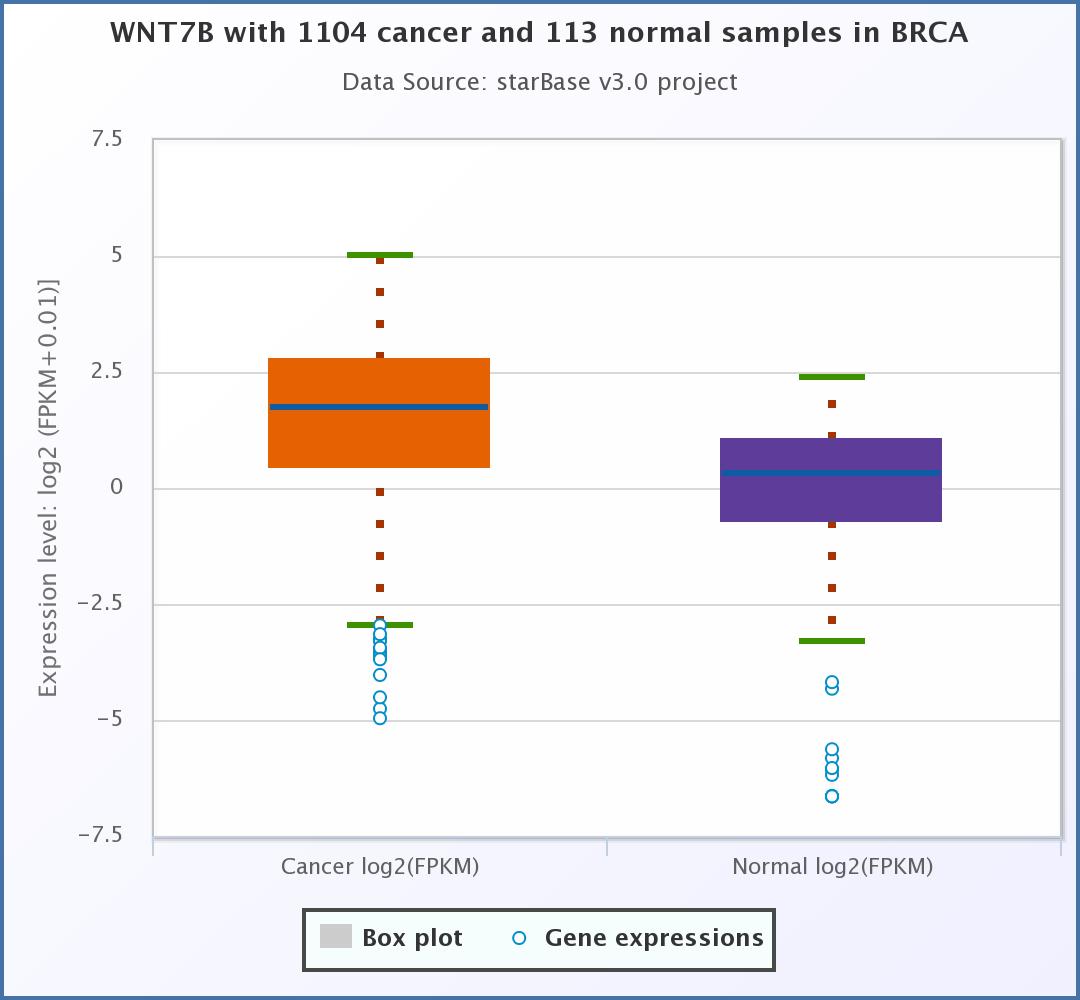

Supplement: Supplementary Figure 1 — Expression of Wnt7b in BC (TCGA database). [file Image_1.jpeg]
