# Supplementary material for: The Role of miR-640: A Potential Suppressor in Breast Cancer via Wnt7b/β-catenin Signaling Pathway
Source: Front Oncol. 2021 Apr 12;11:645682. doi: 10.3389/fonc.2021.645682 (PMC8072343; doi:10.3389/fonc.2021.645682)
Supplement: Supplementary file 2 [file Table_1.docx]

**Table S1 Primers used in this study**

| **Gene** | **Sequences** |
| --- | --- |
| miR-640-F | 5’-GCCCCTGCAGAGCACTGCGG-3’ |
| miR-640-F | 5’-GGCCACCCGGCGGCCGGCAA-3’ |
| U6-F  U6-R  GAPDH-F  GAPDH-R | 5’-CAAATTCGTGAAGCGTTCCATAT-3’  5’-GCTTCACGAATTTGCGTGTCATCCTTGC-3’  5’-CAGGAGGCATTGCTGATGAT-3’  5’-GAAGGCTGGGGCTCATTT-3’ |
| Wnt7b-F  Wnt7b-R | 5’-ATGCACAGAAACTTTCGCAA-3’  5’-TGCATCCGGTCCTCTAGAAC -3’ |
| β-Actin-F | 5’-AGAAAATCTGGCACCACACC-3’ |
| β-Actin-R | 5’- GGGGTGTTGAAGGTCTCAAA-3’ |
